# Paternal Filicide in Sweden: Background, Risk Factors and the Cinderella Effect

**DOI:** 10.1177/14747049241265623

**Published:** 2024-08-28

**Authors:** Hans Temrin

**Affiliations:** 1 Division of Ethology, Department of Zoology, 7675 Stockholm University, Stockholm, Sweden

**Keywords:** paternal filicide, risk factors for filicide, Cinderella effect, filicide in Sweden

## Abstract

An overrepresentation of stepchildren as victims of filicide has been explained as a consequence of ‘discriminative parental solicitude’. The idea being that Darwinian selection has favoured parental love and concern only for biological children, and when such parental feelings are absent, as in stepparents, conflicts with a child could easier escalate to lethal violence. An alternative explanation for this overrepresentation of stepchildren is that risk factors for filicide, such as criminal behaviour and mental health problems, are more prevalent in stepparents. This study focused on paternal filicide in Sweden and investigated (i) if stepchildren are overrepresented as victims of filicide compared with biological children, (ii) if filicides are committed in a context that implies a ‘conflict with the child victim’ and (iii) if stepfathers and biological fathers differ in characteristics associated with filicide risk. The analyses showed that stepchildren were overrepresented as victims compared with children of fathers in families with two biological parents and this overrepresentation was even higher in young children. Children of single biological fathers and children of non-residential biological fathers were also overrepresented as victims of filicide. Less than 20 percent of the filicides were committed in the context of a ‘conflict with the child’ and in these cases only stepchildren were overrepresented as victims. In the population at large, both stepfathers and single biological fathers had higher rates of mental health problems, violent criminality and illegal possession of drugs compared with fathers in families with two biological parents.

## Introduction

Relatively high rates of abuse and lethal violence against stepchildren have been reported in a number of studies, with the reasons being widely discussed (e.g. Adler-Baeder, 2006; Buller, 2005; Daly & Perry, 2022; Daly & Wilson, 1988, 2008; Giles-Sims & Finkelhor, 1984; Nobes et al., 2019; Schacht et al., 2021). One explanation is based on an evolutionary perspective on human psychology. The idea being that Darwinian selection has favoured psychological mechanisms which efficiently target investment in children in relation to a parent's genetic interest. The strong feelings of love and concern parents experience toward their own children ensure that they invest in their best genetic interest. It has been suggested that these affectionate parental feelings should mitigate conflicts and decrease the risk for lethal violence against a child. When such feelings are absent, which would then be most expected in stepparents, the risk for child abuse and lethal violence could increase (Burgess & Draper, 1989; Daly & Wilson, 1980, 1988, 2008). This higher risk of violence towards stepchildren has been named the Cinderella effect, and having a stepparent, it is claimed, is the most powerful risk factor for severe child maltreatment yet discovered (Daly and Wilson, 1998, 2008). Although this discriminative parental solicitude in favour of parents’ ‘own children’ is considered an adaptation (Daly & Wilson, 1988, p. 83), the killing of a stepchild is not since it is a rare phenomenon that does not enhance the killer's average fitness (Daly & Perry, 2022).

The first study to empirically investigate child abuse in an evolutionary context, focusing on the genetic relationship between perpetrator and victim, was Wilson et al. (1980) who analysed risks of child abuse in different family types. They showed that the risk was highest in families of single biological fathers, while children in stepfamilies had the second highest risk and children in families with two biological parents had the lowest risk. The authors suggested that the reason for this overrepresentation of child abuse by single biological fathers was ‘…that men left alone with children (and especially infants, in which group the relative risk of the father-only household was especially high) are not well prepared – whether emotionally or otherwise – to care for them adequately’ (Wilson et al. 1980, p. 338). They also found that single biological mothers were overrepresented in cases of child abuse, and this was explained as being a consequence of their relative poverty. Wilson et al. (1980) then suggested that different factors explained the high risk of child abuse in single biological fathers and in single biological mothers. The high risk of child abuse found in stepfamilies was explained as following the evolutionary prediction that substitute parents would care less for children when the caretaker is not genetically related to a child. It was concluded that this followed the rule in nature where ‘discriminative attachment and ruthless treatment of alien young have evolved in various forms’ (Daly & Wilson 1980, p. 279).

Other researchers have suggested that the association between stepparents and child maltreatment could be a result of other characteristics of parents in stepfamilies that also increase the risk for maltreatment. Giles-Sims & Finkelhor (1984) suggested that adults who have impulse control problems or a tendency to use violence are less likely to stay in a relationship and would therefore be more likely to be found in stepfamilies. Hence, adults with existing dysfunctions would, on average, be more likely to both enter into stepfamilies and to have characteristics that increase the risk for child maltreatment compared to parents in families with two genetic parents (Finkelhor et al. 2007; Giles-Sims & Finkelhor, 1984; Turner et al., 2007). Since parents are not randomly assigned into family types, the reasons behind why a parent has ended up in a specific family type should to some extent reflect that individual's background and personality. Such differences between parents in different family types could then confound a test for an effect based upon the non-genetic relationship between perpetrator and victim. Therefore**,** it is important to investigate if characteristics that are known to increase the risk for child maltreatment are more common among stepparents than among biological parents.

Early studies of child abuse, which focused on their multidimensional character, reported that abusers often had previous criminal convictions and personality characteristics of anxiety, depression and irritability (Fergusson et al., 1972; Gil, 1969). The abusers also had a lower educational level than average and were more often unemployed (Gil, 1969). Studies of child maltreatment have established an association between mental health problems and risks for lethal violence in a number of studies (e.g. D’Orban, 1979; Nordlund & Temrin, 2007; Resnick, 1969; Sidebotham & Golding, 2001). Therefore, if mental health problems are relatively more common in certain family types this could increase their risk for lethal violence against children. Another risk factor for severe child maltreatment is if parents have a history of violent criminality (Stanley & Goddard, 2004). Temrin et al. (2011) showed that parents in stepfamilies, both among filicide perpetrators and in the population of Sweden, had, on average, more records of crimes in general and of violent crimes than parents in families with two biological parents. A third risk factor for child maltreatment is drug abuse, which in a worldwide survey was found to be a common and serious factor associated with lethal violence against children (UNICEF, 2003).

One aim of this study is to investigate if these three risk factors, i.e. mental health problems, a history of violent criminality and a history of drug abuse, are unevenly distributed between family types. Another aim is to investigate motive and background to lethal violence against children. The evolutionary hypothesis is that a non-genetic relationship between parent and child increases the risk for lethal violence because conflicts with a child will be more likely to escalate when affectionate feelings are absent.

If parental homicides – especially those occurring beyond the child's infancy - result largely from temporary, angry lapses of parental solicitude, then the variable magnitude of child-specific parental love is likely to influence the risk of such lapses. … Children annoy adults frequently, and the risk that the adult might react so angrily as to damage the child must surely be influenced by the particular adult's degree of concern for the particular child's welfare. (Daly & Wilson, 1988, p. 75).

Hence, to be able to predict higher risks for children based upon relatively little parental love, lethal violence against a child should primarily be the result of a conflict with the child victim.

The aims of this study are to investigate (i) if stepchildren are overrepresented as victims of filicide compared with biological children, (ii) if filicides are committed in a context that implies a ‘conflict with the child’ – here defined as a filicide primarily targeted towards the child victim with the intent to change the child's behaviour by the use of force or to punish the child, and (iii) if stepfathers and biological fathers differ in characteristics associated with filicide risk. To investigate if the filicide risk was higher for stepchildren than for biological children, all filicides in Sweden during 1965–2009 were analysed. Filicide is defined here as the killing of a child under the age of 15 years by its parent or by its stepparent. Only filicides by fathers were included in the analyses since almost all stepparents in the data set were stepfathers. Children in four different family situations were included in the study: children in families with two biological parents, children in families with a single biological father, children who lived with single biological mothers but were killed by their non-residential father, and stepchildren. To achieve a more complete picture of patterns of filicide in relation to genetic relationship between parent and child, single biological parents were also included in the comparisons. To investigate whether filicides were committed in the context of a conflict with the child, the description of the crimes and the cause of the filicide as stated in the death certificate were analysed. To investigate if risk factors for filicide were unevenly distributed between stepfathers and biological fathers, criminal records of fathers in the population of Sweden were analysed together with data on mental health problems.

## Methods

### The Filicides

The data set involves the solved cases of filicide in Sweden from the start of 1965 to the end of 2009. Filicide is defined here as the killing of a biological child by the biological parent or the killing of a stepchild by the stepparent. In this study**, **only children killed by biological fathers or stepfathers are included. Statistics Sweden provided a death certificate of each individual child who was killed during the study period. From this data set, the filicide cases were identified with the help of police records and court sentences. The police investigations of the individual cases were collected at local police stations and the psychiatric evaluations at the Department of Forensic Psychiatry, Huddinge Hospital **in** Stockholm, Sweden. A case was considered solved if the police had identified the killer and/or if the perpetrator had been convicted. In this study, the children were less than 15 years old, which was also the age of children from earlier studies of child homicide in Sweden (Somander and Rammer, 1991; Sturup & Granath, 2015). There were three children in the data set taken from Statistics Sweden of victims of filicide that were 15 years old (and therefore not included in this study). All three were killed by fathers in families with two biological parents.

### Children in Different Family Types

Statistics Sweden provided data on the number of children living in each family type based on nationwide surveys of the years 1985 and 1990. The average of these two years was then used in the analyses, since this is close to the mid-year of the filicide data (1965–2009), for number of children living in the different family types.

The four categories of children in the study are stepchildren living with a stepfather and a biological mother; children living with a single biological father; children living in a family with two biological parents; and children who lived with their single mothers but were killed by their non-residential biological father.

### Mental Health Problems among Parents in Sweden

Data on mental health problems among parents in Sweden are based on interviews carried out by Statistics Sweden on samples of parents in different family types during the years 2000–2009. These were the years for which it was possible to obtain such data. Parents were classified according to whether they suffered from any mental health problems and their answers were classified by Statistics Sweden according to the International Classification of Diseases Version 9 (ICD-9). The diagnoses considered included schizophrenia, psychosis, depression, nervous problems, stress and intellectual disability.

### Criminality among Perpetrators

The Swedish National Council for Crime Prevention provided data on previous crimes committed by the filicide perpetrators in this study. Two categories of crimes were included here: ‘violent crimes’ and ‘illegal possession of drugs’. Violent crimes were defined as being directed towards other adults and included murder, manslaughter, assault, robbery and rape. The filicides and any earlier physical child abuse were not included here. ‘Illegal possession of drugs’ includes selling, buying, producing and possession of narcotic drugs.

### Criminality among Parents in Sweden

The data on criminality in Sweden were ordered from Statistics Sweden and are based on databases for the years 1985 and 1990 on violent crimes and possession of illegal drugs crimes for all parents in Sweden. Violent crimes include murder, manslaughter, assault, robbery and rape. ‘Illegal possession of drugs’ includes selling, buying, producing and possession of narcotic drugs. The data from Statistics Sweden gives the number of parents (with children < 18 years) with criminal convictions in different family types. Each person is only counted once for each category of crime, independently of the number of crimes for which they had been convicted. The mean values of the frequencies of the years 1985 and 1990 are used in the analyses, since that is close to the mid-year 1987 of the period 1965–2009 for the data on filicide.

In an earlier study of filicide risk and criminality in Sweden (Temrin et al., 2011), filicides of parents in stepfamilies (irrespective of whether the perpetrator was the biological parent or the stepparent in the stepfamily) were compared with filicides of parents in families with two biological parents. In this study, only paternal filicide is studied and filicides committed by the stepfather of the victim are compared with filicides by biological fathers in several family types. This study also differs from Temrin et al. (2011) in the respect that both criminality and mental health problems among fathers in three different family types in the population at large are compared.

### Motive and Background of the Filicides

The classification of the context of a filicide is based on the motive and background described in the police investigation of the individual cases, the court documents and the psychiatric evaluations of perpetrators. Some filicides in the data set had too limited information on background and context of the crimes to be included in these analyses. Three classifications were used: ‘conflict with the child’, ‘conflict with the mother’ and ‘mental health problems’. Note that a filicide could be included in more than one of these categories.

The filicides classified as being committed in a ‘conflict with the child victim’ were primarily the ones targeted towards the child victim in the context of a conflict with the child and with intent to change the child's behaviour by use of force. This was usually to stop the child from crying or to punish the child. In addition to this, there was one case included into this category where the child was killed by the father immediately after birth, two cases where the fathers were classified as being in a psychotic state when beating the child victims to death, and one case where the young child victim died due to drugs and rape.

Perpetrators were classified into the category ‘mental health problems’ if the background material stated that they had been diagnosed with mental health problems before the filicides were committed. I did not include classifications of the mental health of the perpetrators made after the crime was committed.

The perpetrators in the category ‘conflict with the mother’ were those where the filicide was described as being committed in the context of a conflict with the mother of the child victim. For instance, when the perpetrator and the mother were in a custody conflict or when the filicide was described as being committed due to a divorce or separation.

### Cause of Death

Cause of death was taken from the death certificate of each child victim provided by Statistics Sweden. From this, the method used by the perpetrator to kill the child was supplemented by the use of police records and court sentences. The 21 filicides classified as ‘beatings’ were in 18 cases where the perpetrator beat or hit the child with their hand, in one case using a hammer, in one case using a wood log and in one case by throwing the child against the wall in the room.

### Statistical Methods

Goodness-of-fit tests were used for the statistical analyses of the data in Tables 1, 3, 6 and 7. The expected proportions were calculated from number of children in a certain family type (Tables 1 and 3) or the number of fathers in each family type in Sweden (Table 6). In the analyses in Table 7, the expected proportions were calculated from the number of parents interviewed in each family type. All statistical tests were conducted in SPSS version 29. Holm's sequential Bonferroni procedure was used to calculate the adjusted *p*-values in the pairwise comparisons in Tables 1, 6 and 7 (Abdi, 2010; Quinn & Keough, 2002).

## Results

### Were Stepchildren Overrepresented as Victims of Filicide Compared with Biological Children?

Filicide risk was highest for children of single biological fathers, second highest for stepchildren and lowest for children of fathers in families with two biological parents (hereafter shortened to ‘fathers in 2B’) (Table 1). Thirty-two children who lived with single biological mothers were killed by their biological father who was not living with them. There is no official estimate of the number of fathers in this family situation, but if we make the assumption that all children living with their single biological mother sometimes meet their biological father, the risk for a child living with its single mother and being killed by the non-residential biological father did not differ significantly from the risk for stepchildren to be killed by stepfathers (Table 1). Not all children of single mothers meet their biological father so this assumption will overestimate the number of non-residential fathers that meet their child.

**Table 1.** Filicide Victims of Fathers in Families with two Biological Parents (2B), of Single Biological Fathers (1B), of Stepfathers (S) and of Children Living with Their Single Biological Mother but Killed by Their non-Residential Biological Father (NBF).

Perpetrators	Fathers in 2B (2B)	Single Biological Fathers (1B)	Stepfathers (S)	Non-resident Biological Father (NBF)	All
Number of victims	114	13	15	32	174
*Percentage of victims in family type*	*65.5%*	*7.5%*	*8.6%*	*18.4%*	*100%*
*Children in the population* a	*1,165,468*	*24,176*	*71,101*	*172,638* b	*1,433,383*
*Percentage children in family type in population*	*81.3%*	*1.7%*	*5.0%*	*12.0%*	*100%*
Victims in relation to children in Sweden (% victims / % children in population)	0.81	4.41	1.72	1.53	

Exact goodness-of-fit: *p* = .0000004, df = 3. Pairwise comparisons (*p*-values of Holm's sequential Bonferroni procedure within parentheses): 2B-1B *p* = .000002 (.000012); 2B-S *p* = .0069 (.0207); 1B-S *p* = .0157 (.0314); 2B-NBF *p* = .0018 (.0090); 1B-NBF *p* = .0023 (.0092); NBF-S *p* = .7484. All df = 1.

^a^ Mean of 1985 and 1990 of children in Sweden.

^b^ Number of children living with their single biological mother.

If we split the data set in two in relation to age of the victims, the filicide risk for the youngest children (<5 years) was 10 times higher for children of single biological fathers and almost six times higher for stepchildren compared with the risk for children of fathers in 2B. The risk for a child (<5 years), who lived with its single biological mother, to be killed by its non-residential biological father was more than double the risk for a child of a father in 2B (see Appendix 3 for more information).

### Were Filicides Committed in a Context That Implies a ‘Conflict with the Child Victim’?

The motive and background of a filicide was classified to investigate whether the fatal actions were committed primarily in the context of a conflict with the child or if the victims were killed in other contexts. A ‘conflict with the child’ would be, for instance, that the perpetrator intended to stop a child's behaviour (like crying or being disobedient, see Methods). A non-conflictual filicide would be, for instance, the intentional suffocation of a sleeping infant or a father shooting the child victim in an extended suicide.

Seventeen percent of the filicide victims were killed in the category ‘conflict with the child’. In stepchildren, most of the victims (53%) were killed in this context. The corresponding figures were 18% of the victims killed by fathers in 2B, four percent of the victims killed by their non-residential biological father, while no victim of single biological fathers was killed in the context of ‘a conflict with the child’ (Table 2). Twenty of the 24 victims killed in this category were beaten to death, one victim was stabbed with a knife, one was killed with a hammer, one victim was shaken to death and one victim died from injuries of being raped and given narcotic drugs. Only one victim was killed in each case in this category of filicides.

**Table 2.** the Contexts of the Filicides in Different Family Types.

Victims killed^a^	‘Conflict’ with the Child	Mental Health Problems^b^	Conflict with the Mother
Fathers in 2B	17.9% 15/84	41.0% 34/83	50.6% 42/83
Single biological fathers	0 0/13	61.5% 8/13	61.5% 8/13
Stepfather	53.3% 8/15	33.3% 5/15	53.3% 8/15
Non-resident biological fathers	3.6% 1/28	32.1% 9/28	85.7% 24/28
*Fisher-Freeman-Halton Exact Test*	*p* *=* *.0003*	*p* *=* *.3300*	*p* *=* *.0080*
All	17.1% 24/140	40.3% 56/139	59.0% 82/139

^a^ A child victim could be in more than one category.

^b^ Mental health problems are those diagnosed before the filicides were committed.

If we restrict the analyses to the filicides committed in the category ‘conflict with the child’, only stepchildren were overrepresented as victims (Table 3).

**Table 3.** Filicides in the Different Family Types When Committed in ‘a Conflict with the Child Victim’.

Perpetrators	Fathers in 2B	Single Biological Fathers	Stepfathers	Non-residential Biological Fathers^a^	All
Number of victims	15	0	8	1	24
Victims in relation to children in population (% victims / % children in population, see Table 1).	0.77	0	6.17	0.35	
	Biological fathers	Stepfathers			
Number of victims	16 *66.7%*	8 *33.3%*	*Exact goodness of fit p* *=* *.00002*		
*Children in Sweden*	*94.6%*	*5.4%*			
Victims in relation to children in population	0.71	6.17			

^a^ Child victim lived with their single biological mother.

The majority of victims (59%) were killed in the context of a conflict between the perpetrator and the mother of the victim. The rate was 86% in non-residential biological fathers and 51–62% in the other three categories of fathers (Table 2). Three of the seven single biological fathers who committed filicide in the context of a conflict with the mother of the child were in a custody conflict, while the other four perpetrators committed filicide due to jealousy or a depression after a separation/divorce. No single biological father killed the mother of the child. In non-residential biological fathers, almost all filicides were committed in the context of a custody conflict or due to jealousy or depression after a separation/divorce. Six of the 19 perpetrators who killed in this category also killed the mother of the child. Of the seven stepfathers who committed filicide in the context of a ‘conflict with the mother of the child’, three of the stepfathers killed due to the partner/wife wanting separation/divorce, one due to depression and three in the context of a fierce quarrel. Two stepfathers killed the mother of the child. Among fathers in 2B, 17 of the 26 perpetrators who killed ‘in a conflict with the mother’ did so due to their partner wanting separation/divorce. The other nine of the 26 perpetrators committed filicide in the context of fierce quarrel. Eleven of the 26 perpetrators in 2B also killed their spouse/partner.

Forty percent of the child victims were killed by perpetrators that had been diagnosed having ‘mental health problems’ before the filicides were committed. There was no significant difference in this respect between fathers in the four family types (Table 2).

Suicide (fatal and non-fatal) occurred in 55% (24/44) of the perpetrators in the category ‘mental health problems’, in 66% (39/59) of the perpetrators in the category ‘conflict with the mother’, while there was no suicide among the perpetrators in the category ‘conflict with the child’ (0/24). The suicide rate for stepfathers was 14%, for fathers in 2B 58%, for single biological fathers 72% and for non-residential fathers 77% (details in Appendix 2). Suicide rate was significantly lower in stepfathers compared with biological fathers [14.3% (2/14) vs. 63.2% (74/117); Fisher's Exact Test: *p* = .0008, Odds ratio = 0.097, 95% CI = 0.021–0.453]. In each case where there was background information, the father had planned the filicide-suicide.

The cause of death for each child victim was stated in the death certificate. The five most common causes were, in order, gunshot, stabbed with knife, strangled, beaten to death and killed by gas (stove/car) (Table 4). Together, 76% of the victims were killed in these ways. When the child was killed by gunshot, knife, being strangled or killed by gas, then the intention was to kill the child. In the cases of the victim being beaten to death, it was usually an act to stop the child from crying or with the attempt to discipline the child, and not with the intent to kill the child. A higher proportion of stepfathers beat their victims to death compared with biological fathers [33.3% (5/15) vs. 10.1% (16/159); Fisher's Exact Test: *p* = .0212, Odds ratio = 4.469, 95% CI = 1.358–14.710; data from Table 4]. Suicide did not occur in any of the perpetrators who beat a child to death and not more than one child was killed in each case.

**Table 4.** Causes of Death for Child Victims of Fathers in Four Family Situations.

Means Used in Filicide	*All*	Father in 2B	Single Biological Father	Stepfather	Non-resident Biological Father
Gunshot	***22.4%*** *39*	**22.8%** 26	**23.1%** 3	**13.3%** 2	**25.0%** 8
Knife	***15.5%*** *27*	**14.0%** 16	**23.1%** 3	**20.0%** 3	**15.6%** 5
Strangled	***15.5%*** *27*	**14.0%** 16	**15.4%** 2	**13.3%** 2	**21.9%** 7
Beaten	***12.1%*** *21*	**13.2%** 15^a^	**0** 0	**33.3%** 5	**3.1%** 1
Gas (stove/car)	***10.9%*** *19*	**14.0%** 16	**7.7%** 1	**0** 0	**6.3%** 2
Killed by ax/hammer/frying pan	***5.2%*** *9*	**4.4%** 5	**0** 0	**13.3%** 2	**6.3%** 2
Arson	***4.6%*** *8*	**3.5%** 4	**15.4%** 2	**0** 0	**6.3%** 2
Drowned	***4.0%*** *7*	**3.5%** 4	**7.7%** 1	**0** 0	**6.3%** 2
Poison (drugs)	***2.9%*** *5*	**3.5%** 4	**0** 0	**6.7%** 1	**0** 0
Suffocated	***1.2%*** *2*	**0** 0	**7.7%** 1	**0** 0	**3.1%** 1
Hanged	***1.2%*** *2*	**1.8%** 2	**0** 0	**0** 0	**0** 0
Other methods	***4.6%*** *8*	**5.3%** 6	**0** 0	**0** 0	**6.3%** 2
Total	***100%*** *174*	**100%** 114	**100%** 13	**100%** 15	**100%** 32

^a^ Including one child shaken to death.

‘Other methods’ included:

**For victim of fathers in 2B:** Two victims were thrown from a balcony, two victims were killed in a car crash in an extended suicide, one victim was thrown to the floor, and one victim was killed by a train in an extended suicide. For one victim there was no data on method.

**For victims of non-residential biological fathers:** One child victim was killed by an explosion in an extended suicide, and one victim was killed by a train in an extended suicide.

### Did Stepfathers and Biological Fathers Differ in Characteristics Associated with Filicide Risk?

Among the perpetrators, stepfathers had more records of earlier violent criminality than biological fathers (Table 5; Odds ratio = 7.091, 95% CI: 1.710–29.412).

**Table 5.** Earlier Records of Violent Criminality for Perpetrators in Different Family Types.

Perpetrators	Earlier Record of Violent Criminality^a^	
Fathers in 2B	15.9% 7/44	17.5% 11/63
Single biological fathers	12.5% 1/8	
Non-residential biological fathers	13.6% 3/22	
Stepfathers	60.0% 6/10	60.0% 6/10
	*Fisher-Freeman-Halton Exact Test p* *=* *.0206*	*Fisher exact test p* *=* *.0082*

^a^ For definition of violent criminality, see Methods.

Those who killed in a conflict with the child victim had higher rates of earlier violent criminality (50%; 8/16) than the perpetrators who committed filicide in other contexts (14.8%; 9/61) (Odds ratio = 5.778, 95% CI: 1.725–19.352).

**Table 6.** Criminal Records of Fathers in Different Family Types in Sweden.

Criminal Records *Means of 1985 and 1990*	Fathers in 2B	Single Biological Fathers (1B)	Stepfathers (S)	*Chi-square* *Test (Goodness-of-fit)*
Violent crimes	3.47% *26,031*	7.72% *2075*	7.78% *6157*	*χ^2^* *=* *4273.834* *p* *<* *.0001* *df* *=* *2*
Illegal possession of drugs	0.71% *5322*	2.09% *563*	1.89% *1494*	*χ^2^* *=* *1640.373* *p* *<* *.0001* *df* *=* *2*
Number in population	*87.61%* *750,107*	*3.14%* *26,892*	*9.25%* *79,182*	*100%* *856,181*

Chi-square (Goodness-of-fit), pairwise comparisons, *p*-values of Holm's Bonferroni correction within parentheses: Violent crimes: 2B-1B χ^2^ = 1294.793, *p* < .0001 (<.0001); 2B-S χ^2^ = 3418.646, *p* < .0001 (<.001); 1B-S χ^2^ = 0.090, *p* = .765 (.765). Illegal possession of drugs: 2B-1B χ^2^ = 657.015, *p* < .0001 (<.0001); 2B-S χ^2^ = 1207.224, *p* < .0001 (<.0001); 1B-S χ^2^ = 4.435, *p* = .035 (.035). All df = 1.

In the population at large, records of violent crimes were more common among stepfathers and single biological fathers than among fathers in 2B. There was no significant difference between stepfathers and single biological fathers in this respect. (Table 6). Illegal possession of drugs was slightly more common among single biological fathers than stepfathers, and least common among fathers in 2B (Table 6).

Single fathers had the highest proportion of anxiety-related problems, with stepfathers second and fathers in 2B having least problems (Table 7). Psychiatric disorder was more common in single fathers than in fathers in 2B, while all other differences were non-significant (Table 7).

**Table 7.** Mental Health Problems of Fathers in Different Family Types in the Population of Sweden.

Mental Health Problems (years 2000–2009)	Fathers in 2B	Single Biological Fathers (1B)	Stepfathers (S)	*Chi-square* *Test (Goodness-of-fit)*
Anxiety-related problems	13.21% *825*	22.38% *130*	16.87% *136*	*χ^2^* *=* *35.473, p* *<* *.0001* *df* *=* *2*
Psychiatric disorder (ICD-9) of long duration	2.40% *150*	4.13% *24*	2.73% *22*	*χ^2^* *=* *6.287, p* *=* *.0431* *df* *=* *2*
Parents interviewed	*81.83%* *6247*	*7.61%* *581*	*10.56%* *806*	*100%* *7634*

Chi-square (Goodness-of-fit), pairwise comparisons, *p*-values of Holm's Bonferroni correction within parentheses: Mild problems of nervousness, uneasiness and anxiety: 2B-1B χ^2^ = =31.936, *p* < .0001 (<.0001); 2B-S χ^2^ = =7.033, *p* = .0080 (0.016); 1B-S χ^2^ = 5.327, *p* = .0210 (0.0210). Psychiatric disorder: 2B-1B χ^2^ = 6.238, *p* = .0125 (0.0375); 2B-S χ^2^ = 0.315, *p* = .5749 (0.5749); 1B-S χ^2^ = 1.999, *p* = .1575 (0.3150). All: df = 1.

## Discussion

### Were Stepchildren Overrepresented as Victims of Filicide Compared with Biological Children?

As predicted by the evolutionary hypothesis of the Cinderella effect, stepchildren were overrepresented as victims of stepfathers compared to children killed by fathers in families with two biological parents. The risk was more than doubled for stepchildren of all ages (<15 years) while the risk for young stepchildren (<5 years) was almost six times the risk of being killed by fathers in families with two biological parents. The children most at risk for filicide were those living with their single biological father. Also, the risk for a child to be killed by its non-residential biological father was higher than the risk for children of fathers in families with two biological parents. Temrin et al. (2000, 2004) found no significant overrepresentation of stepchildren as victims of filicide compared to children in families with two biological children, while this study of paternal filicide for a longer time period shows a clear overrepresentation of stepchildren as victims of filicide in Sweden.

One must be cautious to draw general conclusions about filicide from a study of filicide in one country for a restricted time period, since it is likely that countries differ in the circumstances that increase or decrease the risk for filicide. For evolutionary hypothesis of why the filicide risk of single parents and stepparents in Nordic countries should differ from the risk in other countries and concerning why we should expect a higher proportion of mental health problems among filicide perpetrators in Nordic countries, see Ottesen (2023).

### Were Filicides Committed in a Context That Implies a ‘Conflict with the Child Victim’?

To show that a higher risk of filicide for stepchildren is due to non-genetic relatedness, filicide must not only be more prevalent in stepchildren than in biological children, but also the cause of the overrepresentation must be consistent with the evolutionary explanation. The evolutionary hypothesis of the Cinderella effect on filicide is based on two assumptions: (i) that parents who are not genetically related to a child will give them less parental love and care than biological parents would, and (ii) that less parental love leads to more lethal conflicts with a child (Daly & Wilson, 1988). That non-genetic relationship parent and child means less parental care has been shown in stepparents (e.g. Amato, 1987; Anderson et al., 1999a, 1999b; Hofferth & Anderson, 2001), but not in adopted children (e.g. Gibson, 2009; Segal et al., 2015). Second, that less parental love leads to more lethal conflicts with a child is difficult to investigate and has, as far as I know, not been investigated. What we can investigate is if a conflict with the child victim is an important background to filicide. In this study, 17% of the filicides were committed in the context of a conflict with the child victim and 12% of the victims were beaten to death. In a study of paternal filicide in the Netherlands, 27% of the victims were classified as ‘battered child’ (Liem & Koenraadt, 2008) and in a study of over 900 filicides in Canada, 27% of them were classified as ‘beating/blows’ (Dawson, 2015). Another study of filicide, this time only on young victims (<5 years) in the United States, a much higher proportion of the filicides were lethal beatings (Weekes-Shackelford & Shackelford, 2004). However, even if filicides in general are not committed in the context of a conflict with the child victim, filicide could still be more likely committed by parents who experience little parental love and concern towards their children.

The background of the filicides differed in several respects between the perpetrators in the different family types. In stepfathers, most filicides had a background in a ‘conflict with the child victim’, while relatively few of the filicides committed by single fathers, fathers in families with two biological parents and non-residential biological fathers were committed in that context. This, together with the fact that lethal beatings were also more common in stepfathers than in biological fathers, show that filicides by biological fathers and stepfathers took place in different contexts and for different reasons. When the analyses of filicide rates were restricted only to the filicides committed in a conflict with the child, only stepchildren were overrepresented as victims. That a relatively high proportion of stepfathers who committed filicide fatally beat their victims to death and were in conflict with the child follows the results of Daly and Wilson (1994), Weekes-Shackelford and Shackelford (2004), Harris et al. (2007) and Nobes et al. (2019), who all showed that filicide by stepparents were disproportionately likely to involve abuse and death by beatings. These findings are also consistent with the evolutionary prediction of relatively high risks of lethal conflicts between stepparents and stepchildren. It is also consistent with the idea that other factors, like a violent personality, are more common in stepfathers. In this study, stepfathers who committed filicide had a significantly higher frequency of earlier violent criminality than biological fathers. This was also suggested in an Australian study where more than half of the male perpetrators had been convicted for a prior offence (specifically violent crimes), and 74% of the stepparents had a history of criminality (Brown et al., 2019).

One study of child abuse has tried to investigate if ‘controlling for antisociality would eliminate the stepfather effect’ (Hilton et al., 2015, p. 9). All men in their study had an earlier police report of domestic violence and the researchers tested whether different degrees of severity of earlier domestic violence predicted risks of child abuse in stepchildren and in biological children. The analyses showed that among men who all had records of earlier domestic violence, and who had both stepchildren and biological children in the family, the odds of assaulting a stepchild at different levels of antisociality were twice the odds of assaulting a biological child (Hilton et al., 2015).

This study is on filicide and not on child abuse. The proportion of filicides that have a history of child abuse varies considerable between studies. For instance, Sidebotham et al. (2011) reported that 22% of the filicides in their data set from England were in the category ‘severe physical assaults’. In Finland, Putkonen et al. (2011) reported that 34% of filicidal fathers had physically abused their victims earlier. Daly and Wilson (2008, p. 386) state that fatal battering is very different from filicide-suicide by a depressed parent ‘…who may construe killing her children as a “rescue”…’. If anything, we should expect ‘conflicts with the child’ to be more prevalent in physical child abuse since that is an act primarily directed towards a specific child, while a substantial proportion of filicide perpetrators have motives primarily directed toward themselves or the mother of the child victim (for a review see West, 2007).

One indication that a filicide was primarily caused by the perpetrator's own problems and not by a conflict with the child is if it was followed by suicide, and especially a preplanned suicide. In this study, filicide-suicides were always planned by the perpetrator and were never committed in the context of a conflict with the child victim. Suicide (fatal and non-fatal) occurred in 58% of the perpetrators. Similarly, 60% of paternal filicides in Québec were followed by suicide or attempted suicide (Bourget & Gagné, 2005). In a study of filicide committed by both male and female perpetrators, 54% of the perpetrators in Finland and 32% of the perpetrators in Austria attempted suicide or died of suicide at the crime scene (Putkonen et al., 2009). The filicide-suicide rate reported in a study in Chicago was much lower (Shackelford et al. 2005). There was a difference in suicide rate between perpetrators in different family types in this study. Few of the stepfathers but a majority of biological fathers attempted or died of suicide. This difference in suicide rate between stepparents and biological perpetrators corresponds to the findings in Daly and Wilson (1994) and in Harris et al. (2007), while Shackelford et al. (2005) did not find a difference between biological parents and stepparents in this respect.

When filicides in different family types are committed in different contexts, where some indicate conflicts with the child but most have a background in the perpetrator's own problems, like mental health problems, depression due to separation or a severe custody conflict, it would be difficult to predict relative risks of filicide between family types based upon a factor primarily related to parental love.

### Did Stepfathers and Biological Fathers Differ in Characteristics Associated with Filicide Risk?

One explanation for differences in filicide rates between family types is that parents differ in characteristics that increase the risk for filicide. This is supported by the results of this study, where both stepfathers and single biological fathers in the population at large had more records of violent criminality and of illegal possession of drugs than fathers in families with two biological parents. However, even if single biological fathers had more records of violent criminality than fathers in families with two biological parents, no single biological father committed filicide in the context of a conflict with the child victim or beat the child to death. Also, that more than 70% of the filicides by single biological fathers were followed by suicide compared with 14% of the filicides by stepfathers, suggests that the background of filicide differs between the two categories of fathers. The family situation of single fathers is obviously different from the situation of stepfathers in the respect that while stepfathers are primarily in a stepfamily because they were attracted to the mother, single biological fathers are likely to have a strong relationship with their child. The special situation of stepfathers was also pointed out by Daly and Wilson (1988, pp. 84–85) when they explained why they anticipated that non-genetic relation would only increase risks of filicide for stepchildren but not for adopted children; ‘Whereas the adoptive couple specifically desires to establish a fictive parent-offspring relationship, the stepparent will usually have entered into the relationship incidentally to the establishment of a desired mateship’.

This study showed that mental health problems were most common in single biological fathers in Sweden. Differences between family types in levels of mental health problems were also shown in the UK where single biological parents were most likely to have poor mental health, with parents in stepfamilies second, and with least problems among parents in families with two biological parents (Boyle et al., 2009). In filicide perpetrators, the proportion of mental health problems has been shown to be relatively high. Forty percent of the filicides in this study were committed by fathers having a history of mental health problems, which is more than reported in the UK where 27% of the paternal perpetrators of filicide had a ‘lifetime history of mental illness’ (Flynn et al., 2013). Bourget et al. (2007) in Canada reported that 62% of the paternal perpetrators were diagnosed having a psychiatric illness at the time of the filicide.

## Conclusions

In this study of filicide in Sweden, the risk for a child to be killed by its stepfather, its single biological father or its non-residential biological father was higher than the risk for children of fathers in families with two biological parents. Stepfathers more often killed their victims in conflicts with the child and beat their victims to death than biological fathers did. When the analyses were restricted to the filicides committed in the context of a conflict with the child, only stepchildren were overrepresented as victims. Stepfathers who committed filicide had more records of earlier violent criminality which suggest that they were more violent individuals. This study also showed that the risk factors mental health problems, criminality and drug abuse were more common among single biological fathers and stepfathers in the population at large compared with fathers in families with two biological parents. This overrepresentation of several risk factors in these two categories of fathers is likely to increase the risk for filicide in both stepchildren and children of single biological fathers.

## Appendices

**Appendix 1.** The mean age of the perpetrators and victims in different family types.

	Mean age of perpetrator	Mean age of victim
Fathers in 2B	**35.1 years** *(420.79 months, n* *=* *80 sd* *=* *104.149)*	**4.8 years** *(57.44 months, n* *=* *114* *sd* *=* *50.932)*
Single biological fathers	**36.6 years** *(438.91 months, n* *=* *11 sd* *=* *88.133*	**5.3 years** *(63.69 months, n* *=* *13* *sd* *=* *37.304)*
Stepfathers	**33.4 years** *(401.29 months, n* *=* *14* *sd* *=* *79.116)*	**6.7 years** *(80.53 months, n* *=* *15* *sd* *=* *52.641)*
Non-residential biological fathers	**37.2 years** *(446.85 months, n* *=* *26* *sd* *=* *91.530)*	**5.5 years** *(65.75 months, n* *=* *32* *sd* *=* *49.519)*
*Analysis of variance*	*F* *=* *0.823* *p* *=* *.484* *df* *=* *3*	*F* *=* *1.083* *p* *=* *.358* *df* *=* *3*

**Appendix 2.** Suicide rates (fatal + non-fatal) in relation to the context of the filicide in the four categories of fathers.

Perpetrators	‘Conflict’ with the child	Mental health problem	Conflict with the mother	All
Fathers in 2B	**0** 0/15	**44.4%** 12 (9 + 3)/27	**65.4%** 17 (14 + 3)/26	**57.5%** 46 (39 + 7)/80
Single biological fathers	**0** 0/0	**83.3%** 5 (4 + 1)/6	**57.1%** 4 (4 + 0)/7	**72.7%** 8 (7 + 1)/11
Stepfathers	**0** 0/8	**50.0%** 2 (1 + 1)/4	**28.6%** 2 (1 + 1)/7	**14.3**% 2 (1 + 1)/14
Non-resident biological fathers	**0** 0/1	**71.4%** 5 (3 + 2)/7	**84.2%** 16 (11 + 5)/19	**76.9%** 20 (15 + 5)/26
All	**0** 0/24	**54.5%** 24/44	**66.1%** 39/59	**58.0%** 76/131

Note that a perpetrator could be in more than one category.

**Appendix 3.** Filicide victims <5 years and victims 5–14 years of fathers in families with two biological parents (2B), of single biological fathers (1B), of stepfathers (S) and of children living with their single biological mother but killed by their non-residential biological father (NBF).

Perpetrators	Fathers in families with two biol. parents	Single biol. fathers	Stepfathers	Non-residential biol. fathers	
**Victims < 5 years** (0–59 months)					
No. victims (<5 years)	65	7	5	19	96
*Percentage of victims in family type*	67.7%	7.3%	5.2%	19.8%	100%
*Number of children in family type in popul.* a	425,924	4175	5918	49,428	485,445
*Percentage children in family type in popul.*	87.7%	0.9%	1.2%	10.2%	100%
Victims in relation to children in Sweden (% victims / % children in population)	0.77	8.11	4.33	1.94	
*Exact goodness-of-fit: p* *=* *.0000001, df* *=* *3. Pairwise comparisons (p-values of Holm's sequential Bonferroni procedure within parentheses): 2B-1B p* *=* *.000007 (.00004); 2B-S p* *=* *.0026 (.0104); 1B-S p* *=* *.2536; 2B-NBF p* *=* *.0009 (.0045); 1B-NBF p* *=* *.0031 (.0093); NBF-S p* *=* *.1726 All df* *=* *1-*					
**Victims 5–14 years** (60–179 months)					
No. victims^a^ (5–15 years)	49	6	10	13	78
*Percentage of victims in family type*	62.8%	7.7%	12.8%	16.7%	199%
*Number of children in family type in popul.* a	739,544	20,001	65,183	123,210	947,938
*Percentage children in family type in popul.*	78.0%	2.1%	6.9%	13.0%	100%
Victims in relation to children in Sweden (% victims / % children in population)	0.81	3.67	1.86	1.28	
*Exact goodness-of-fit: p* *=* *.0030, df* *=* *3. Pairwise comparisons (p-values of Holm's sequential Bonferroni procedure within parentheses): 2B-1B p* *=* *.0030 (.0180); 2B-S p* *=* *.0194 (.0970); 1B-S p* *=* *.2335;* *2B-NBF p* *=* *.1444; 1B-NBF p* *=* *.0397 (.1588); NBF-S p* *=* *.3855 All df* *=* *1.*					

^a^ Total number of victims during 45 years (1965–2009).

^b^ The mean number of children in the population of the years 1985 and 1990.
